# ACTH-producing adrenocortical carcinoma: an exceedingly rare diagnosis

**DOI:** 10.1186/s43046-024-00229-z

**Published:** 2024-07-15

**Authors:** Miguel Saraiva, Raquel da Inez Correia, Sérgio Xavier Azevedo, José Ricardo Brandão, José Carlos Oliveira, Isabel Palma

**Affiliations:** 1 Department of Endocrinology, Diabetes and Metabolism, Centro Hospitalar Universitário de Santo António, Largo Professor Abel Salazar, 4099-001 Porto, Portugal; 2 Department of General Surgery, Centro Hospitalar Universitário de Santo António, Largo Professor Abel Salazar, 4099-001 Porto, Portugal; 3 Department of Oncology, Centro Hospitalar Universitário de Santo António, Largo Professor Abel Salazar, 4099-001 Porto, Portugal; 4 Department of Pathological Anatomy, Centro Hospitalar Universitário de Santo António, Largo Professor Abel Salazar, 4099-001 Porto, Portugal; 5 Department of Clinical Chemistry, Centro Hospitalar Universitário de Santo António, Largo Professor Abel Salazar, 4099-001 Porto, Portugal

**Keywords:** Adrenocortical carcinoma, Ectopic ACTH secretion, Cushing’s syndrome, Hypercortisolism, Case report

## Abstract

**Background:**

Adrenocortical carcinoma is a very rare endocrinopathy that has a poor prognosis and is frequently associated with ACTH-independent Cushing’s syndrome. Despite having an adrenocortical carcinoma, our patient surprisingly had an ACTH-dependent Cushing’s syndrome.

Case report.

A 26-year-old female presented with Cushing’s syndrome and an abdominal mass. Imaging studies revealed an adrenal mass consistent with a high-grade malignancy. Laboratory workup showed hypercortisolism, hyperandrogenism, and hypokalemia with normal levels of metanephrines. Unexpectedly, her ACTH levels were remarkably elevated. The pathological analysis of a tumor sample was conclusive for adrenocortical carcinoma with immunopositivity for ACTH.

**Conclusions:**

Our patient suffered from an adrenocortical carcinoma that was ectopically producing ACTH. This case emphasizes that physicians should have a broad-minded approach when evaluating cases of rare endocrine malignancies.

## Background

Adrenocortical carcinoma (ACC) consists of a malignant neoplasm arising from the adrenal cortex that typically has a poor prognosis [1]. It is a very rare endocrinopathy, with an estimated incidence of 0.7 to 2 cases per million per year [1, 2]. The age of diagnosis during adulthood typically ranges around the fifth to sixth decade of life, but another peak may be seen in childhood during the first decade of life [1].

This neoplasm may present with symptoms and signs of hormone excess (40 to 60% of patients) or nonspecific symptoms due to tumor growth (about a third of patients) [3]. More rarely, ACC may be incidentally diagnosed through imaging procedures for unrelated health issues [4]. Biochemical evidence of excess adrenocortical hormone secretion is present in up to 70% of the cases, with hypercortisolism being the most common [1]. If hypercortisolism occurs, patients may present with rapidly progressive Cushing’s syndrome―manifestations may include facial plethora, violaceous abdominal striae, easy bruising, proximal muscle weakness, new-onset diabetes mellitus, osteoporosis, hirsutism (in female patients), hypertension, and hypokalemia [1, 5]. The Cushing’s syndrome associated with ACC is characteristically classified as ACTH-independent, as most patients with cortisol-secreting tumors will have a morning level of ACTH of less than 10 pg/mL [1, 6].

On the other hand, ectopic Cushing’s syndrome (ECS) is an infrequent form of ACTH-dependent Cushing’s syndrome that is typically associated with severe hypercortisolism due to adrenal cortex stimulation by ectopic ACTH secretion [7]. ECS characteristically derives from neuroendocrine tumors, which may vary in clinical aggressiveness and location. The most common causes of ECS are bronchial carcinoids, small-cell lung carcinomas, gut neuroendocrine tumors, thymic carcinoids, medullary thyroid carcinomas, and pheochromocytomas/paragangliomas [7]. Other well-established, albeit rare, possible etiologies include breast [8], ovaries [9] and prostate cancers [10], uterine tumors [11], parotid tumors [12], olfactory bulb neuroblastomas [13], sarcomas, and peritoneal [14] and pleural [15] mesotheliomas. Interestingly, other tumoral sites have been exceptionally described, namely the ileum, mesentery [16], liver [17], and the sphenoid sinus [18].

### Case presentation

A previously healthy 26-year-old woman was referred to our department due to a history of weight gain, peripheral edema, hair loss, hirsutism, and acne that had been evolving for the past 8 months. In the last month, she also reported new-onset arterial hypertension and vinous abdominal striae. Her family physician had already asked for an abdominal computed tomography scan that showed a bulky tumor mass arising from the right adrenal gland with areas of necrosis, invaded the ipsilateral kidney and the liver, and had an estimated size of 16.2 × 14.6x20.2cm. On physical examination at the first endocrinology appointment, the patient had an arterial blood pressure of 160/100 mmHg, facial plethora, dorsocervical fat pad (buffalo hump), large vinous striae on the abdomen and thighs, proximal muscle atrophy, and axillary acanthosis nigricans.

Laboratory studies were ordered, and all of the hypercortisolism screening tests were positive: serum cortisol of 36.2 μg/dL after the 1 mg overnight dexamethasone suppression test, 24-h free urinary cortisol of 1260.0 μg (reference range (RR): 4.3–176.0 μg), and late-night salivary cortisol of 4.070,μg/dL (RR: 0–0.208 μg/dL). Unexpectedly, her morning-ACTH serum level was elevated: 351 pg/mL (RR: 9–52 pg/mL; chemiluminescent immunoassay testing by IMMULITE® 2000); we repeated this dosing and the result was consistent: 556pg/mL (electrochemiluminescent immunoassay testing by Roche Cobas® E601). She had a mild hypokalemia of 3.28 mmol/L (RR: 3.50–5.00 mmol/L) and an elevation of all serum androgens, including dehydroepiandrosterone sulfate (916 μg/dL, RR: 35–430 μg/dL). There was no biochemical evidence of primary hyperaldosteronism or elevation of serum or urinary metanephrines. The most relevant lab results are displayed in Table 1.

**Table 1:** Laboratory results

		**Result**	**Reference range**
**Serum morning cortisol** (μg/dL)		40.7	6.2–19.4
**Hypercortisolism screening**	**Serum morning cortisol after 1mg ODST** (μg/dL)	**36.2**	–
	**24-h free urinary cortisol** (μg)	**1260.0**	4.3–176.0
	**Late-night salivary cortisol** (μg/dL)	**4.070**	0–0.208
**Serum morning ACTH** (pg/mL)		**556**	9–52
**Hyperandrogenism screening**	**DHEA-S** (μg/dL)	**916**	35–430
	**Delta-4 androstenedione** (ng/mL)	**16.60**	0.85–2.75
	**3-alpha androstanediol** (ng/mL)	**8.4**	< 4.5
	**Total testosterone** (ng/mL)	**2.16**	0.06–0.82
**Primary hyperaldosteronism screening**	**Active renin** (pg/mL)	182.16	2.79–61.83
	**Aldosterone** (pg/mL)	120.12	13.37–233.55
	**Aldosterone-renin ratio**	0.66	0.52–37.83
**Pheochromocytoma/paraganglioma screening**	**Serum metanephrine** (pmol/L)	147	< 456.3
	**Serum normetanephrine** (pmol/L)	509	< 982.8
	**Urinary metanephrine** (nmol/day)	527	264–1729
	**Urinary normetanephrine** (nmol/day)	1880	480–2424
	**Urinary 3-methoxythyramine** (nmol/day)	1348	0–1447
**Calcitonin** (pg/mL)		< 2.00	0.00–20.00
**Chromogranin A** (ng/mL)		16.1	0.0–100.0
**Sodium** (mmol/L)		**147**	135–145
**Potassium** (mmol/L)		**3.28**	3.50–5.00
**HbA1c** (%)		6.0	4.0–6.0

*ACTH* Adrenocorticotropic hormone, *DHEA-S* Dehydroepiandrosterone sulfate, *HbA1c* Glycated hemoglobin, *ODST* Overnight dexamethasone suppression test

**Fig. 1:**
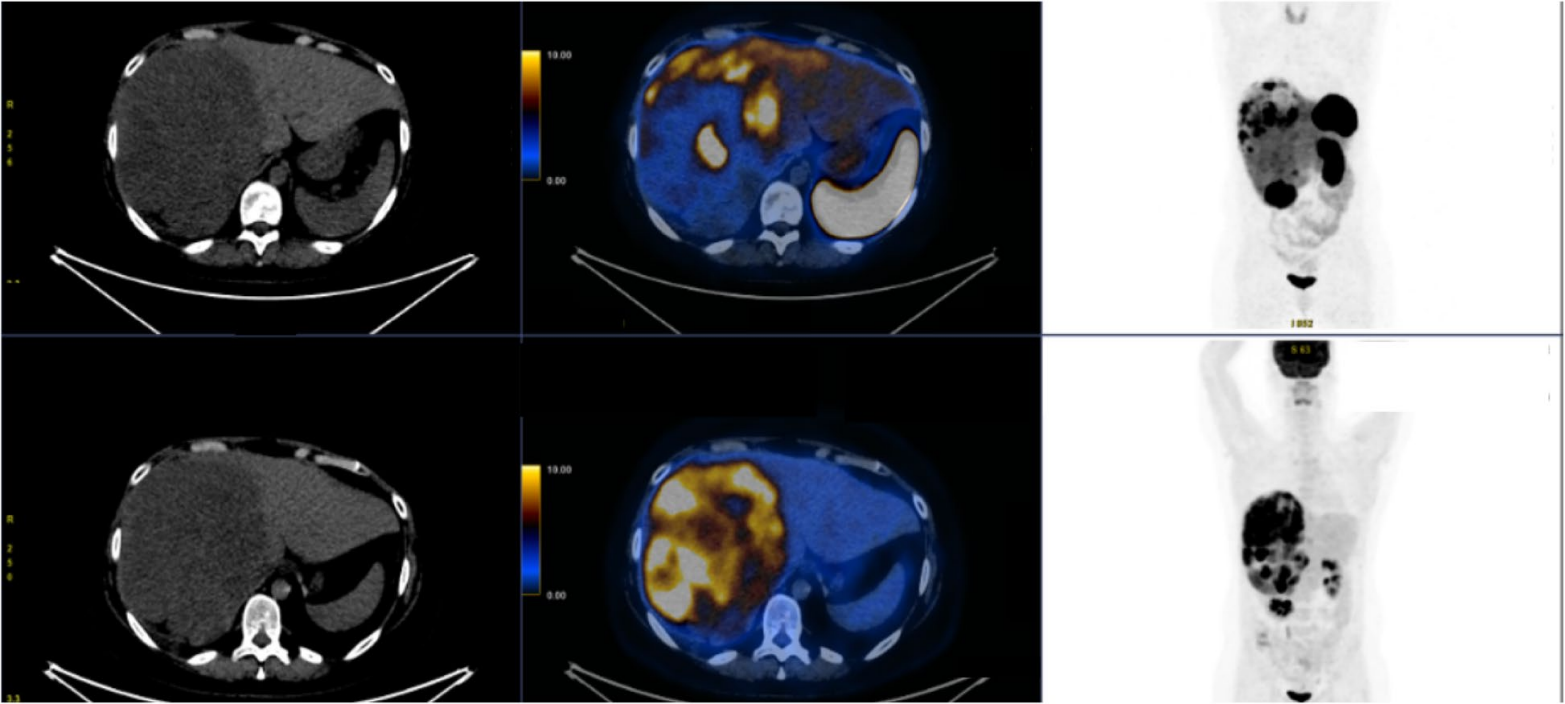
^68^Ga-DOTATOC PET/CT scan showing a bulky adrenal mass and liver metastasis

Functional imaging studies were also performed. A ^18^F-FDG PET/CT scan confirmed an adrenal voluminous hypermetabolic mass (maximum standardized uptake value (SUVmax) of 30.4), compatible with a high-grade malignant neoplasm with invasion of the right lobe of the liver and the right kidney; secondary hypermetabolic liver lesions, suggesting metastasis, were also documented. A ^68^Ga-DOTATOC PET/CT scan further confirmed the aforementioned adrenal mass and liver metastasis (Fig. 1).

Based on these clinical, analytical, and imaging studies, we were able to conclude that the patient probably had a functional ACC causing both hypercortisolism and hyperandrogenism. We could not explain the high levels of ACTH as the PET scans showed no other synchronous tumors that could potentially be associated with ectopic ACTH production.

**Fig. 2:**
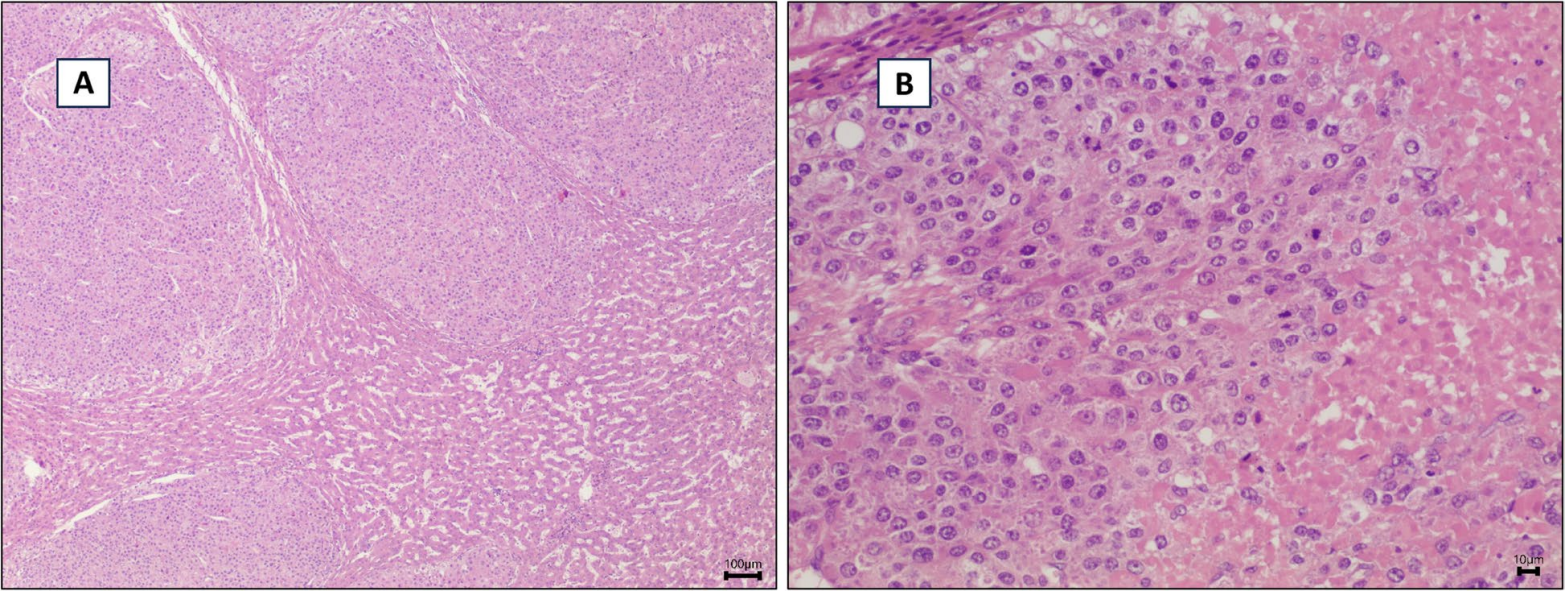
Biopsy from liver metastasis showing infiltration of the liver parenchyma by large nodules of cells with eosinophilic cytoplasm (H&E × 40). Higher power view showing the neoplastic cells have abundant eosinophilic cytoplasm and numerous mitotic figures (H&E × 200)

**Fig. 3:**
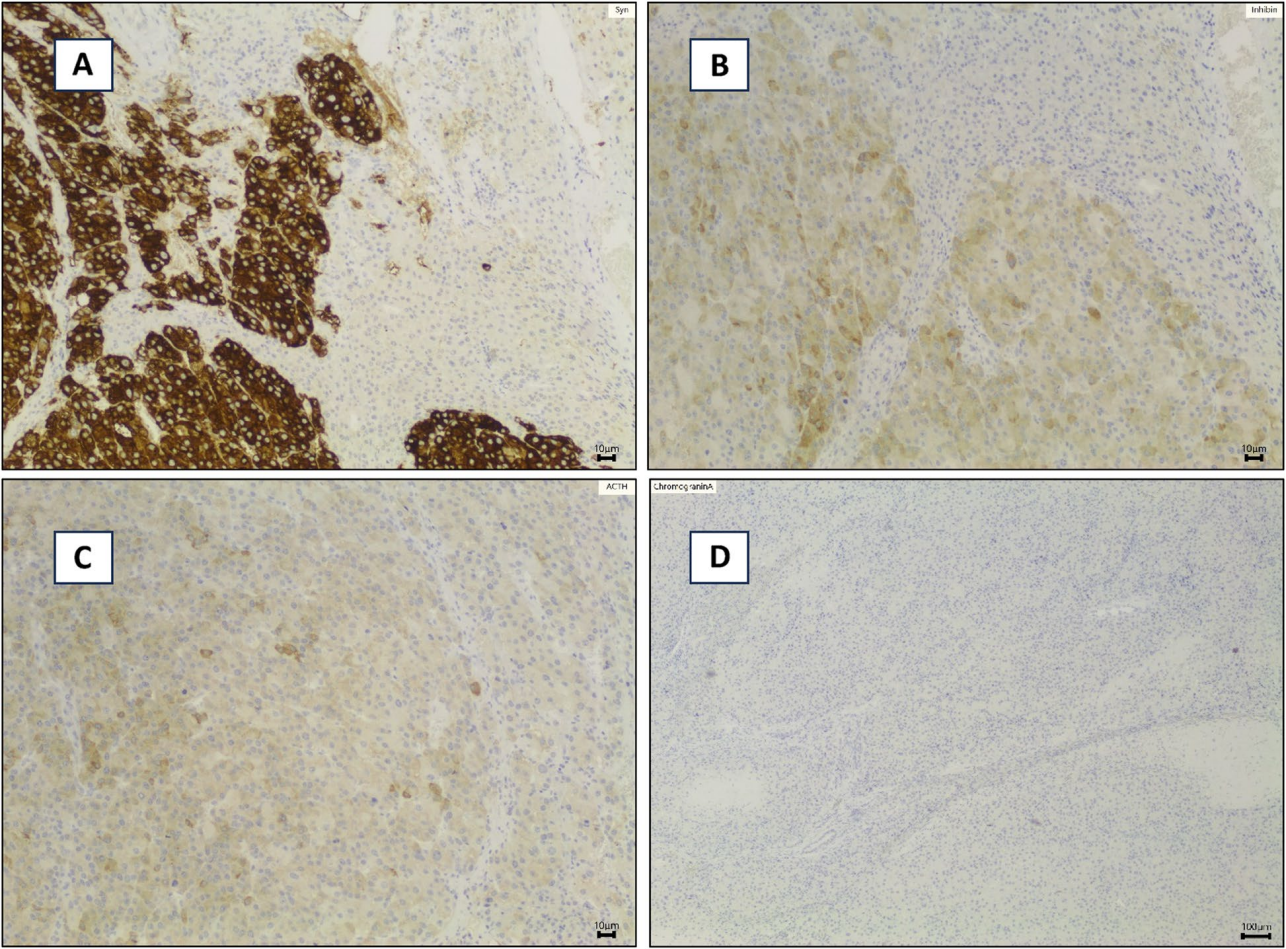
**A** Tumor cells showing positive cytoplasmic staining for synaptophysin (100x). **B** Positive cytoplasmic staining for α-inhibin (100x). **C** Focal positive cytoplasmic for ACTH (100x). **D** Negative staining for chromogranin A (40x)

The patient was then proposed for surgical debulking, but unfortunately during the procedure, the tumor mass was deemed unresectable due to direct invasion of the right kidney and retroperitoneum and the presence of multiple metastatic liver nodules. Additionally, the tumor was subjected to spontaneous rupture during surgery, resulting in the release of tumor tissue into the abdomen. During the procedure, a biopsy from one of hepatic nodules was taken, and histopathological examination revealed infiltration of the liver parenchyma by nodules of large cells with abundant eosinophilic cytoplasm. The neoplastic cells exhibited intranuclear inclusion, brisk mitotic figures (> 20 per 10 high power fields (HPF) visible in the small sample, which is > 20 per 50 HPF according to Weiss score), atypical mitoses, and tumor necrosis (Fig. 2). These findings confirmed the clinical suspicion of metastatic adrenocortical carcinoma to the liver. Immunohistochemical analysis was performed on the liver biopsy, and the tumor cells were found to be positive for synaptophysin, inhibin, and ACTH and were negative for chromogranin (Fig. 3), thus proving the adrenocortical origin of the neoplasm and the ability to synthesize ACTH.

Palliative chemotherapy with a combined regimen of etoposide, doxorubicin, cisplatin, and mitotane was then offered to the patient. She was also started on metyrapone and spironolactone to control both the hypercortisolism and hyperandrogenism symptoms. Unfortunately, the patient ended up suffering from a sudden death event less than a month after her first chemotherapy cycle. The family refused autopsy examination.

## Discussion

In most cases, ACC is associated with clinically relevant adrenal hormone overproduction, with hypercortisolism leading to Cushing’s syndrome being the most frequent form of hormone excess in this setting [3, 4]. Accordingly, our patient displayed both clinical and biochemical evident manifestations of hypercortisolism and met the criteria for Cushing’s syndrome diagnosis. ACC characteristically leads to an ACTH-independent form of this syndrome as the neoplasm arises from the adrenal gland and thus produces cortisol independently from ACTH stimulation [1].

Unexpectedly, our patient had ACTH levels that were remarkably elevated (up to 10.7 times the upper limit of the RR), and this elevation was confirmed by two different laboratory analyzers. We first hypothesized that our patient might have a synchronous occult neuroendocrine tumor producing ACTH or a pituitary corticotropinoma that concomitantly contributed for her exuberant cushingoid features. However, the ^68^Ga-DOTATOC PET/CT scan did not show any evidence of other tumor besides the already known ACC and its metastasis. The hypothesis of an ACTH-producing ACC seemed very unlikely as this neoplasm typically does not produce this hormone. However, immunochemistry examination confirmed that the patient had, in fact, an ACC capable of synthetizing ACTH.

A case published by Dilrukshi et al. [19] was the first and, as far as we know, up until now the only published case of an ACC associated with ectopic ACTH production. Similarly to what happened in Dilrukshi et al.‘s case, in our patient, the immunochemistry examination was important to prove that it was a cortical and not medullary malignant neoplasm (AAC and not pheochromocytoma) and crucial to demonstrate that it was able to produce ACTH. In fact, the immunopositivity for synaptophysin and inhibin and the negative immunoreactivity for chromogranin favors the diagnosis of an ACC. The immunopositivity for ACTH confirms that this tumor was the source of the ACTH aberrant production.

## Conclusion

This case emphasizes that physicians should have a broad-minded approach when evaluating cases of rare endocrine malignancies. ACC may exceedingly rarely produce ACTH and thus may be associated with an ACTH-dependent Cushing’s syndrome and not with the classical ACTH-independent Cushing’s syndrome that typically arises from cortisol-producing adrenal neoplasms.

## Data Availability

Not applicable.

## Abbreviations

ACC: Adrenocortical carcinoma
ECC: Ectopic Cushing’s syndrome
RR: Reference range
